# Supplementary figures and images for: Molecular dynamics study on the strengthening behavior of Delta and Omicron SARS-CoV-2 spike RBD improved receptor-binding affinity
Source: PLoS One. 2022 Nov 17;17(11):e0277745. doi: 10.1371/journal.pone.0277745 (PMC9671323; doi:10.1371/journal.pone.0277745)

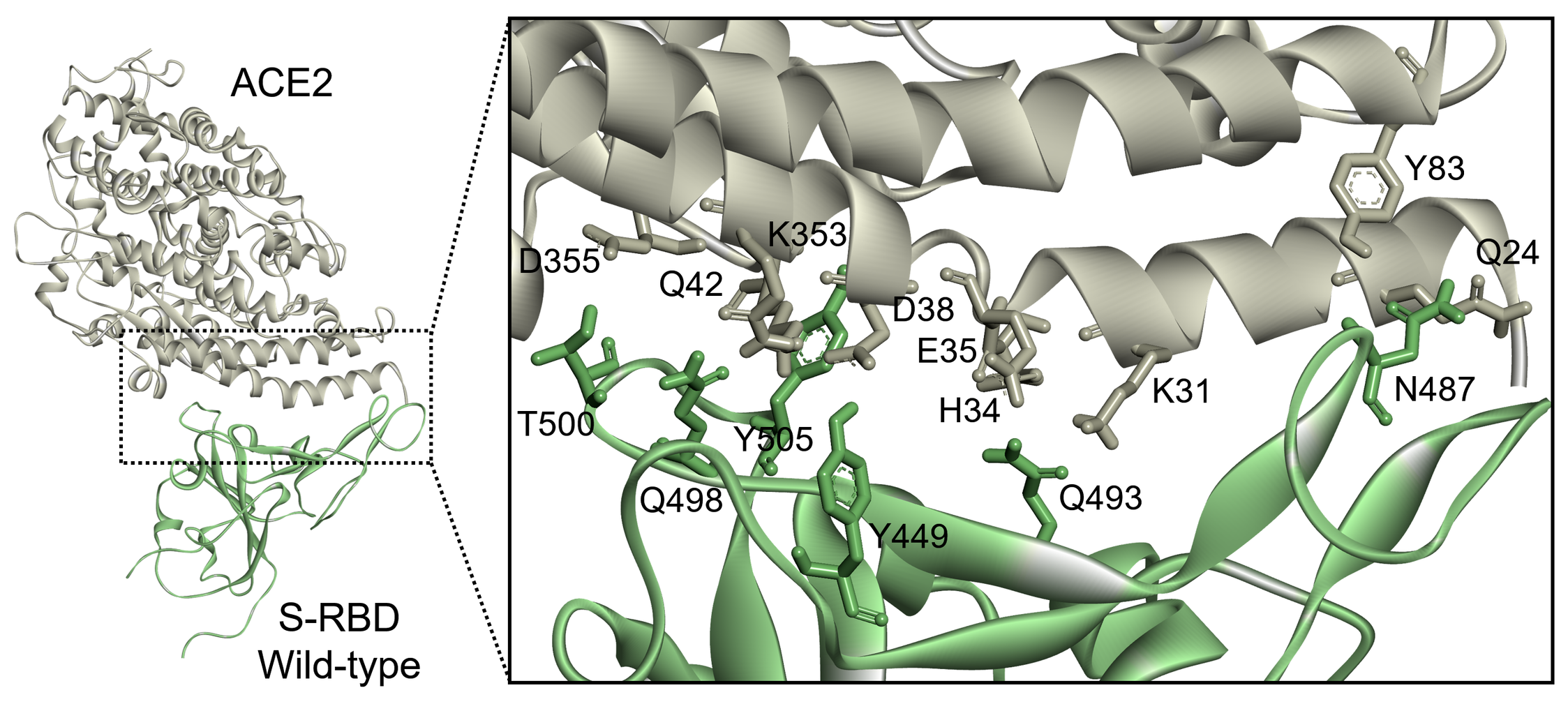

Supplement: S1 Fig — The structure of ACE2 is shown in gray ribbon, while S-RBD is in green ribbon. The close-up of the binding interfaces is shown the crucial hotspots responsible for binding interaction (right panel). The interfacial residues of SARS-CoV-2 S-RBD residues are annotated and shown in green sticks, while ACE2 residues are in gray sticks. The figure was drawn by Discovery Studio 2019 Client (Biovia software). (TIF) [file pone.0277745.s001.tif]

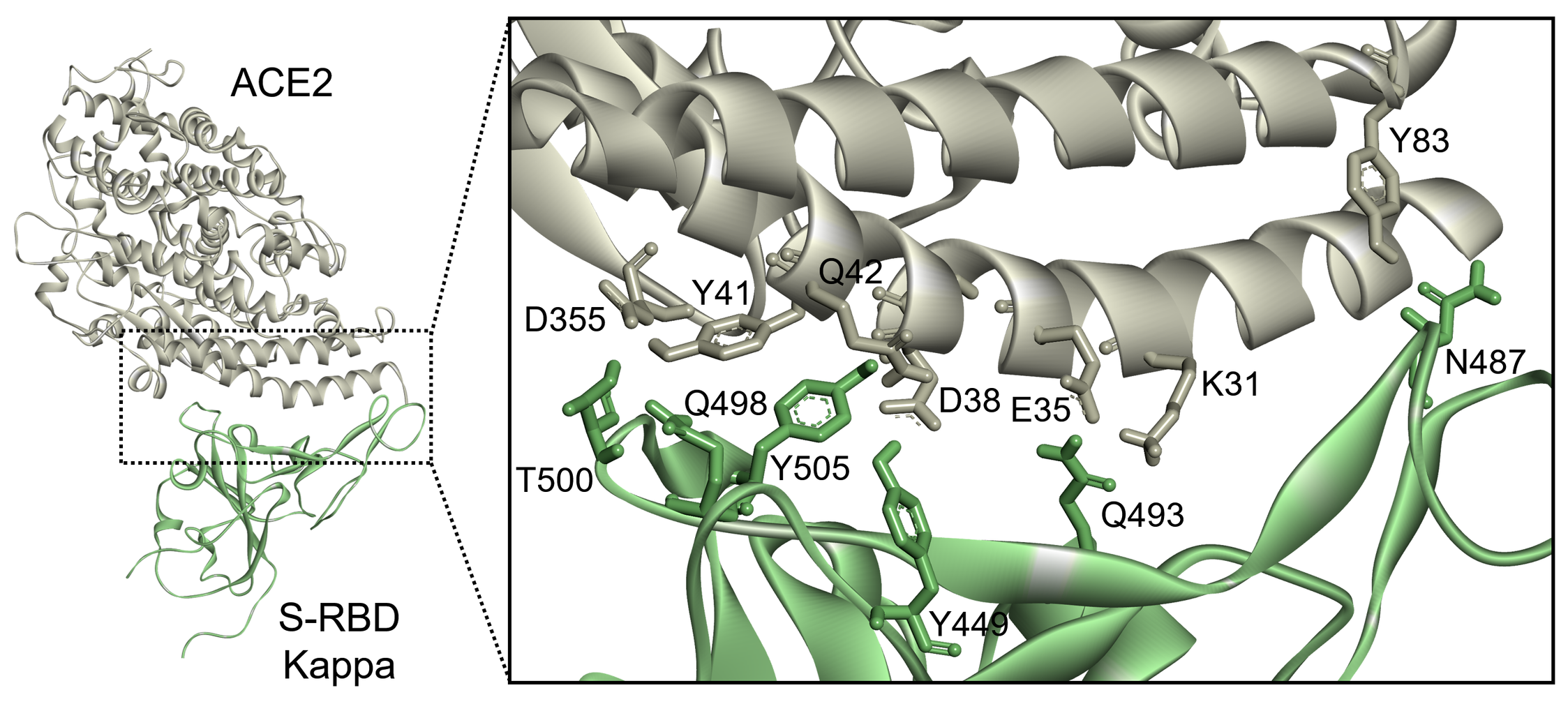

Supplement: S2 Fig — The structure of ACE2 is shown in gray ribbon, while S-RBD is in green ribbon. The close-up of the binding interfaces is shown the crucial hotspots responsible for binding interaction (right panel). The interfacial residues of SARS-CoV-2 S-RBD residues are annotated and shown in green sticks, while ACE2 residues are in gray sticks. The figure was drawn by Discovery Studio 2019 Client (Biovia software). (TIF) [file pone.0277745.s002.tif]

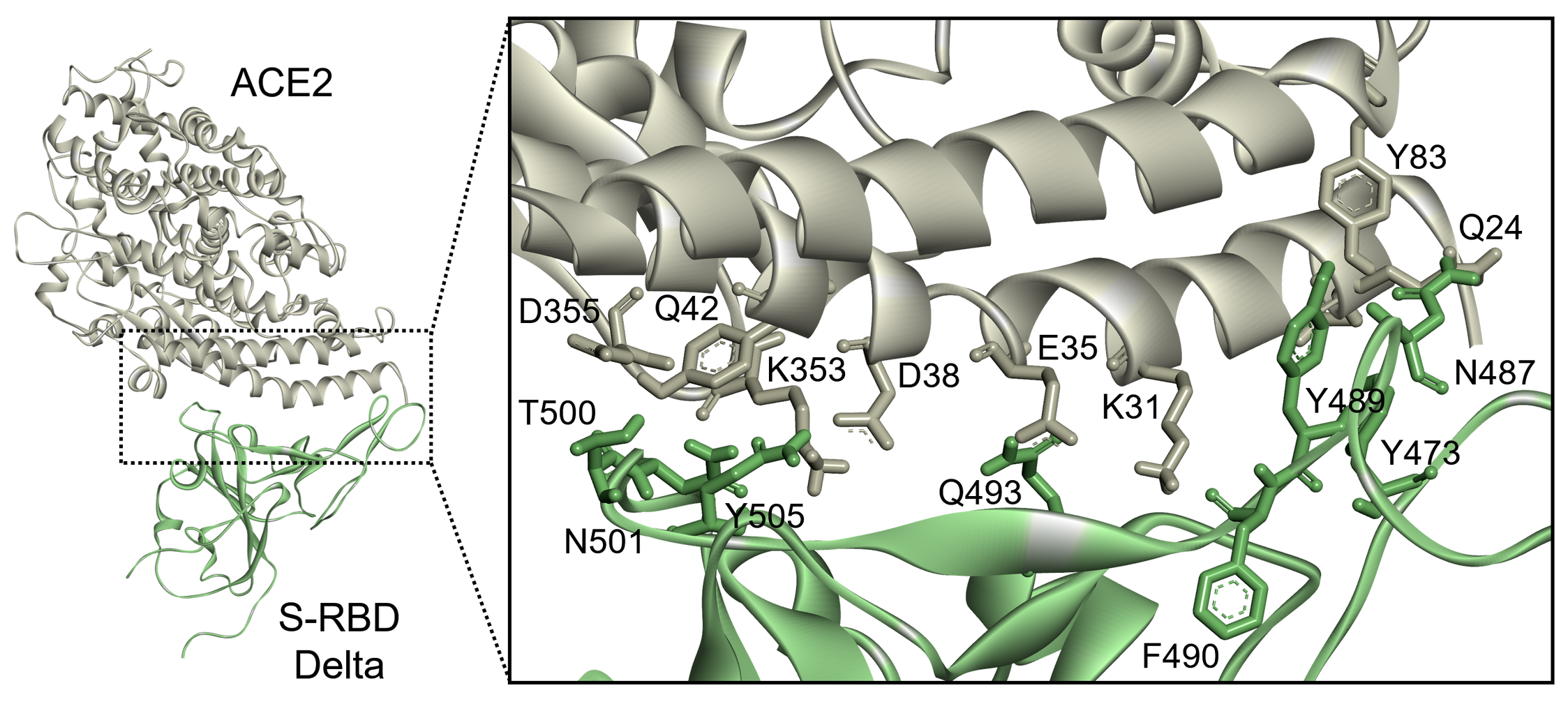

Supplement: S3 Fig — The structure of ACE2 is shown in gray ribbon, while S-RBD is in green ribbon. The close-up of the binding interfaces is shown the crucial hotspots responsible for binding interaction (right panel). The interfacial residues of SARS-CoV-2 S-RBD residues are annotated and shown in green sticks, while ACE2 residues are in gray sticks. The figure was drawn by Discovery Studio 2019 Client (Biovia software). (TIF) [file pone.0277745.s003.tif]

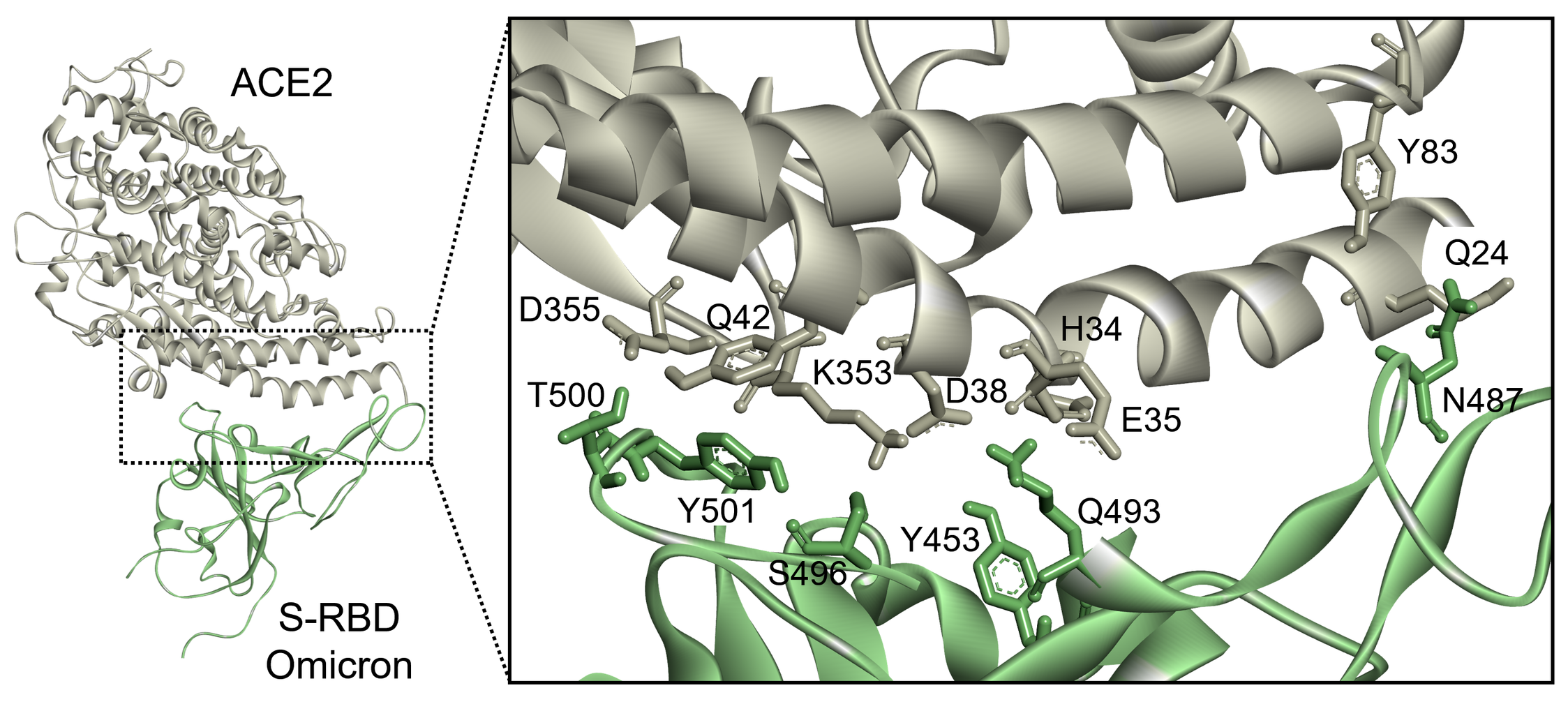

Supplement: S4 Fig — The structure of ACE2 is shown in gray ribbon, while S-RBD is in green ribbon. The close-up of the binding interfaces is shown the crucial hotspots responsible for binding interaction (right panel). The interfacial residues of SARS-CoV-2 S-RBD residues are annotated and shown in green sticks, while ACE2 residues are in gray sticks. The figure was drawn by Discovery Studio 2019 Client (Biovia software). (TIF) [file pone.0277745.s004.tif]

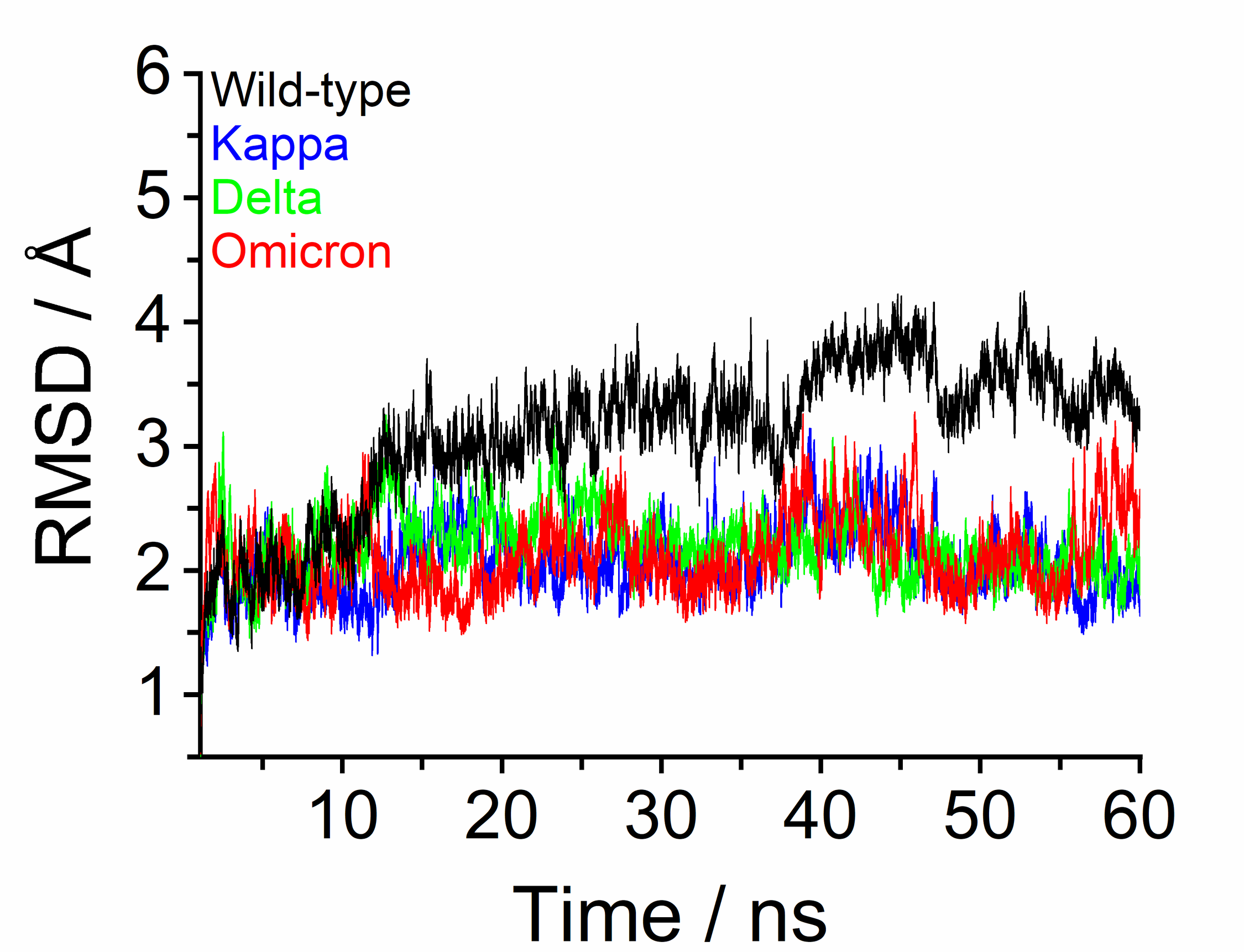

Supplement: S5 Fig — RMSD plot of each S-RBD variant complex with ACE2 protein over the production (Prod) phase MD simulations. (TIF) [file pone.0277745.s005.tif]

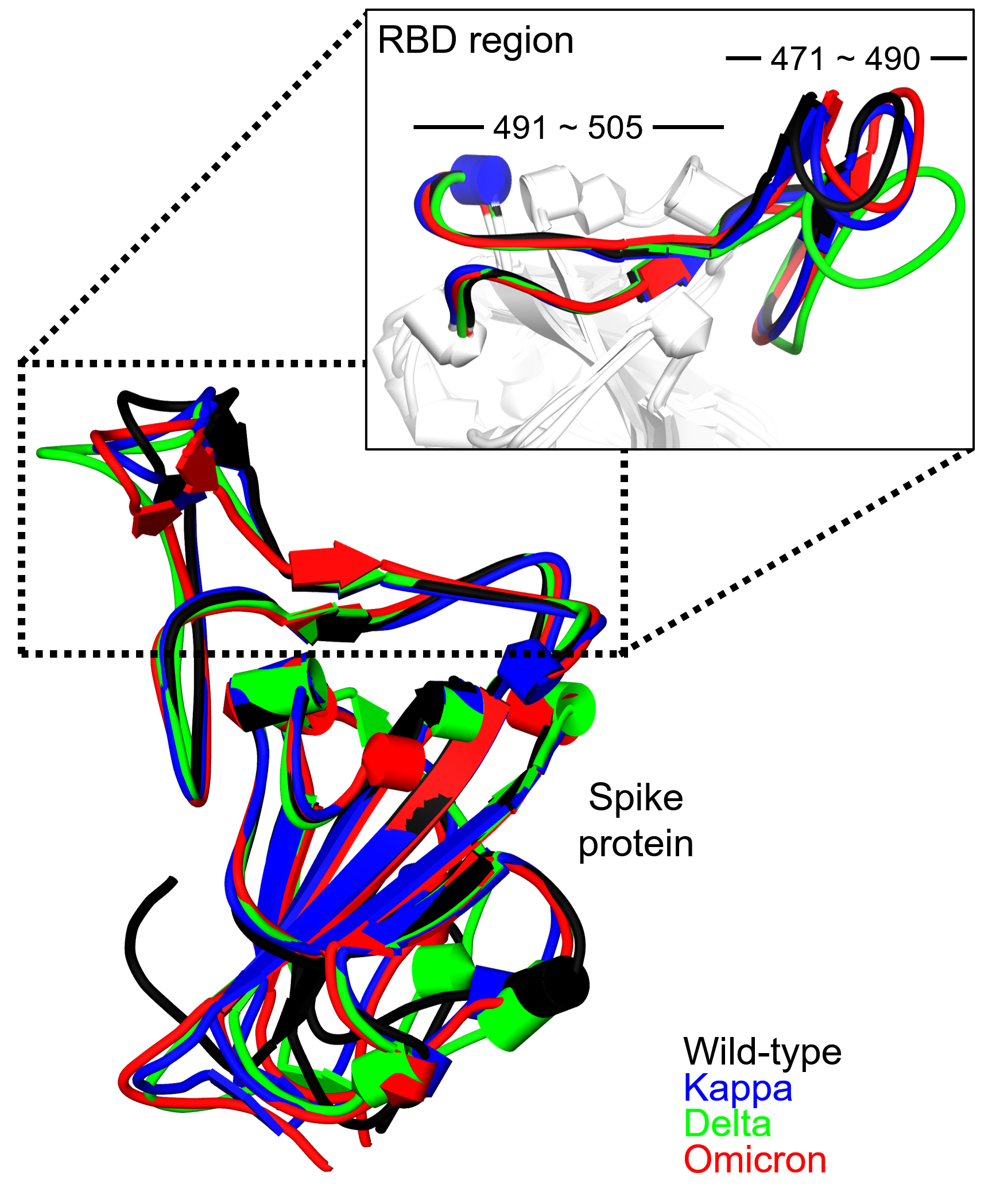

Supplement: S6 Fig — The superimposition structure of the Cα-atom trace of four different structures between the wild-type and mutated S-RBD protein of SARS-CoV-2. The proteins superimpose almost exactly in most parts of the protein expect few regions which exhibit conformation variability (highlighted in the close-up), especially, at the RBD-ACE2 binding interfaces. (TIF) [file pone.0277745.s006.tif]

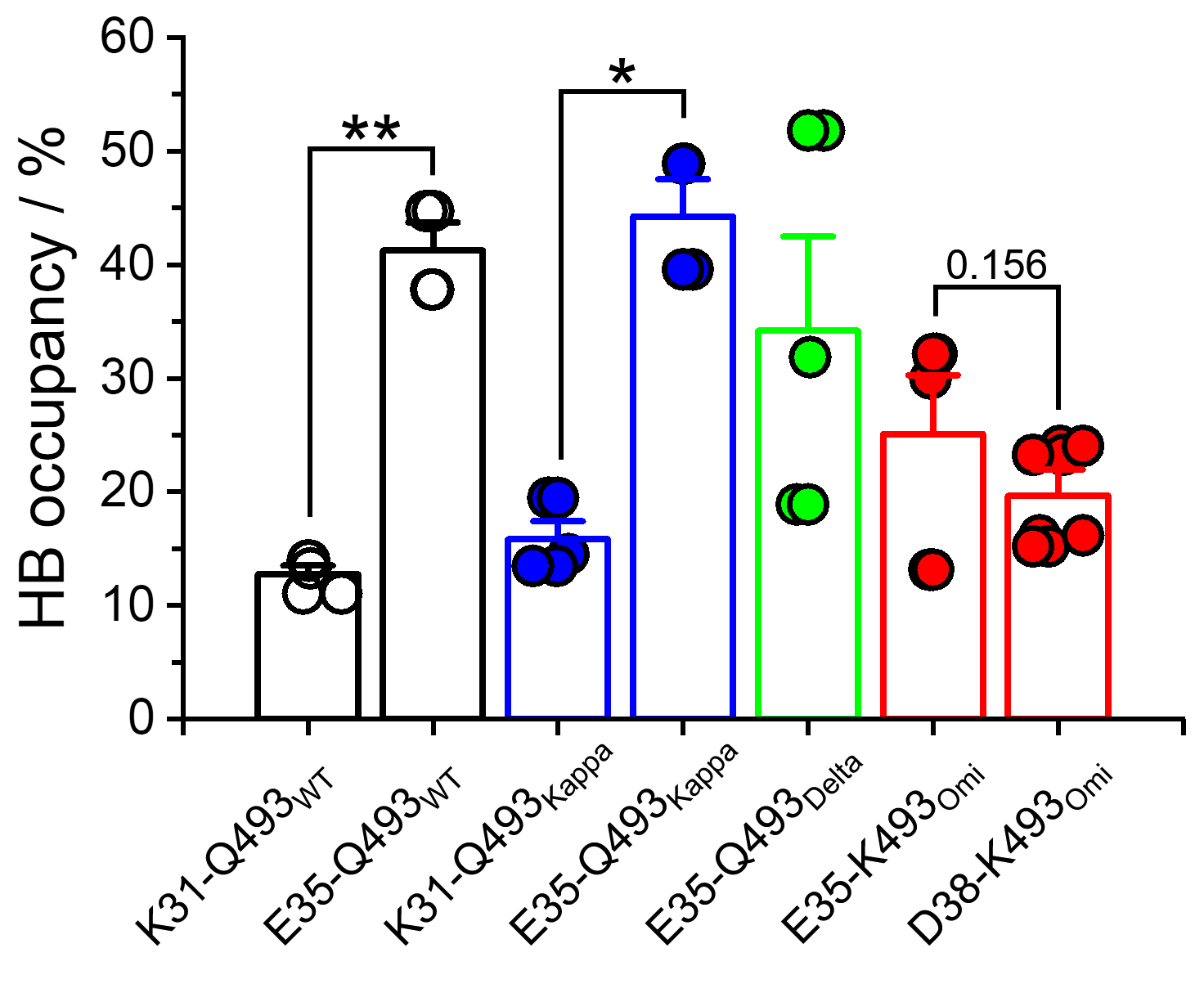

Supplement: S7 Fig — The intermolecular hydrogen bond occupancy between Q493 (or K493 in the Omicron) from S-RBD protein and ACE2 at K31, E35, and D38 for binding complex (* p < 0.05, ** p < 0.005, two-tailed Student’s T-test). (TIF) [file pone.0277745.s007.tif]

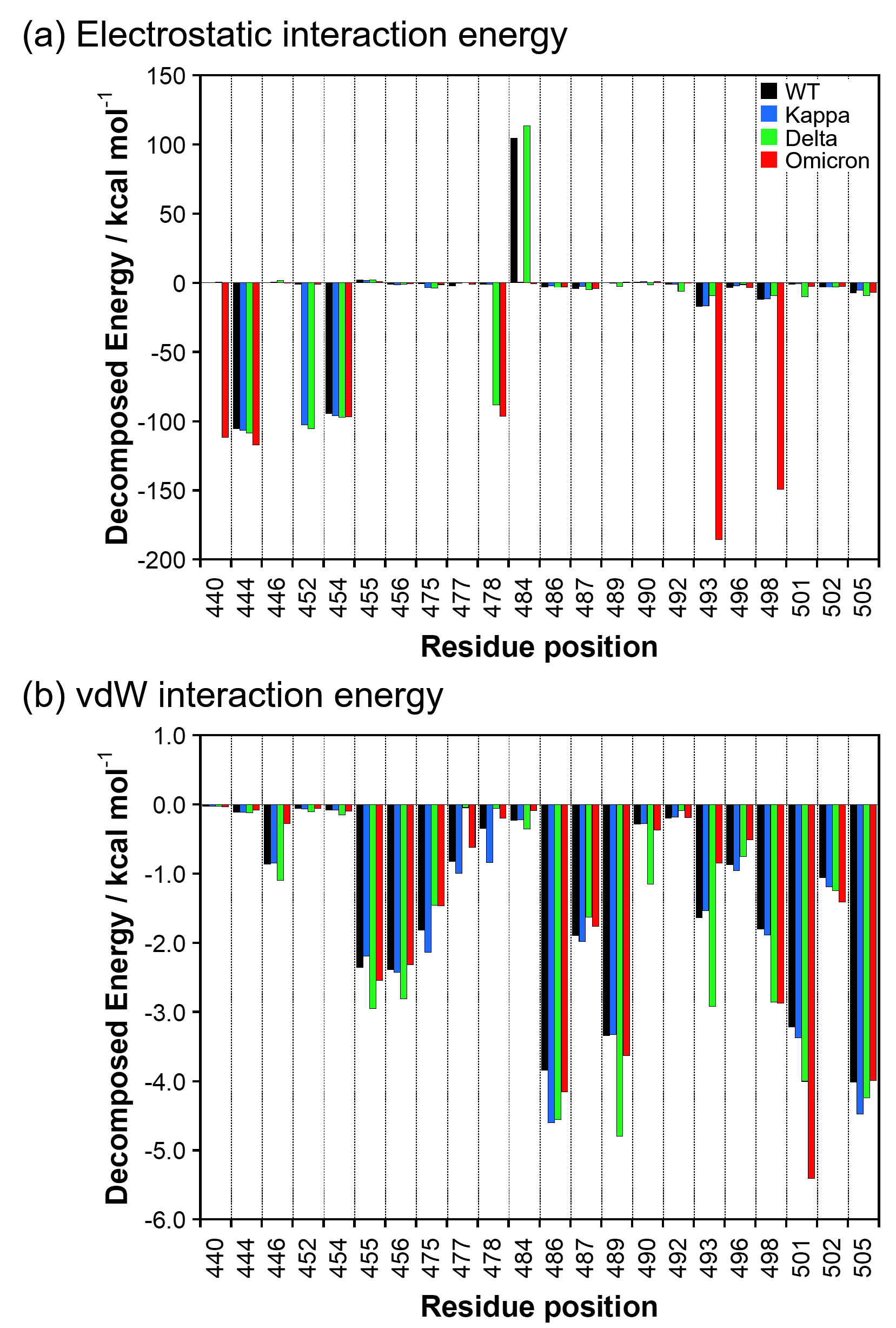

Supplement: S8 Fig — Per-residue decomposed energy of (a) electrostatic, and (b) vdW interaction on the key hotspots of the S-RBD regions (at position 438–505) for ACE2 binding. All values were given in kcal mol-1. (TIF) [file pone.0277745.s008.tif]
