# Supplementary material for: Molecular dynamics study on the strengthening behavior of Delta and Omicron SARS-CoV-2 spike RBD improved receptor-binding affinity
Source: PLoS One. 2022 Nov 17;17(11):e0277745. doi: 10.1371/journal.pone.0277745 (PMC9671323; doi:10.1371/journal.pone.0277745)
Supplement: S1 Table — Relative binding free energy terms (kcal mol–1) calculated by the computational alanine scanning mutagenesis using MM-PBSA method approach for the S-RBD regions (at position 438–505) of SARS-CoV-2 residues effectively involved in the binding interface with the ACE2. (DOCX) [file pone.0277745.s009.docx]

| **Parameters** | **S438A** | **N439A** | **N440A** | **L441A** | **D442A** | **S443A** | **K444A** | **V445A** | **N448A** | **Y449A** |
| --- | --- | --- | --- | --- | --- | --- | --- | --- | --- | --- |
| **ΔΔG*_binding_*** | -0.0847 | -0.0574 | -0.0452 | -0.0054 | 1.7474 | 0.0849 | -2.3007 | -0.0967 | 0.1709 | 1.1784 |
| **ΔΔE*_vdW_*** | -0.004 | -0.103 | -0.012 | -0.009 | -0.009 | -0.020 | -0.038 | -0.099 | -0.030 | -0.417 |
| **ΔΔE*_EEL_*** | 1.523 | -1.037 | 0.437 | 0.361 | 207.693 | 0.575 | -212.131 | -0.061 | -1.187 | -11.768 |
| **ΔΔE*_EPS_*** | -1.604 | 1.082 | -0.470 | -0.357 | -205.936 | -0.471 | 209.869 | 0.065 | 1.387 | 13.610 |
| **ΔΔE*_ENPOLAR_*** | 0 | 0 | 0 | 0 | 0 | 0 | 0 | -0.0013 | 0 | -0.2463 |
| **Parameters** | **N450A** | **Y451A** | **L452A** | **Y453A** | **R454A** | **L455A** | **F456A** | **R457A** | **K458A** | **S459A** |
| **ΔΔG*_binding_*** | -0.0052 | -0.0361 | -0.0944 | 0.7053 | -2.3191 | -2.6739 | -2.6289 | -1.8553 | -1.9664 | -0.0214 |
| **ΔΔE*_vdW_*** | -0.009 | -0.030 | -0.055 | -1.562 | -0.044 | -3.903 | -4.398 | -0.023 | -0.029 | -0.002 |
| **ΔΔE*_EEL_*** | -1.155 | -1.166 | 0.142 | -0.424 | -191.039 | -0.866 | -0.947 | -178.955 | -169.792 | 0.031 |
| **ΔΔE*_EPS_*** | 1.159 | 1.160 | -0.182 | 2.743 | 188.764 | 2.023 | 2.773 | 177.122 | 167.854 | -0.051 |
| **ΔΔE*_ENPOLAR_*** | 0 | 0 | 0 | -0.0513 | 0 | 0.0713 | -0.0571 | 0 | 0 | 0 |
| **Parameters** | **N460A** | **L461A** | **K462A** | **P463A** | **F464A** | **E465A** | **R466A** | **D467A** | **I468A** | **S469A** |
| **ΔΔG*_binding_*** | 0.0380 | -0.3073 | -1.7887 | 0.0080 | -0.4011 | 1.7069 | -1.7950 | 1.8764 | -0.0115 | 0.0192 |
| **ΔΔE*_vdW_*** | -0.013 | -0.008 | -0.003 | -0.002 | -0.003 | -0.003 | -0.005 | -0.005 | -0.004 | -0.002 |
| **ΔΔE*_EEL_*** | 2.830 | -0.214 | -156.762 | 0.083 | -0.076 | 163.510 | -166.447 | 174.845 | 0.139 | 0.982 |
| **ΔΔE*_EPS_*** | -2.779 | -0.086 | 154.976 | -0.074 | -0.322 | -161.800 | 164.657 | -172.964 | -0.147 | -0.961 |
| **ΔΔE*_ENPOLAR_*** | 0 | 0 | 0 | 0 | 0 | 0 | 0 | 0 | 0 | 0 |
| **Parameters** | **T470A** | **E471A** | **I472A** | **Y473A** | **Q474A** | **S477A** | **T478A** | **P479A** | **C480A** | **N481A** |
| **ΔΔG*_binding_*** | -0.0381 | 1.9448 | -0.0542 | -0.1660 | -0.0570 | 1.2090 | -0.1981 | -0.0143 | -0.0366 | -0.0548 |
| **ΔΔE*_vdW_*** | -0.007 | -0.007 | -0.051 | -0.960 | -0.102 | -0.248 | 0.000 | -0.033 | -0.022 | -0.012 |
| **ΔΔE*_EEL_*** | -0.947 | 169.456 | 0.046 | -0.981 | -1.701 | -2.918 | -1.253 | 0.259 | 0.555 | -2.609 |
| **ΔΔE*_EPS_*** | 0.915 | -167.505 | -0.049 | 1.766 | 1.746 | 4.407 | 1.056 | -0.240 | -0.570 | 2.566 |
| **ΔΔE*_ENPOLAR_*** | 0 | 0 | 0 | 0.0086 | 0 | -0.0327 | -0.0011 | 0 | 0 | 0 |
| **Parameters** | **V483A** | **E484A** | **F486A** | **N487A** | **C488A** | **Y489A** | **F490A** | **P491A** | **L492A** | **Q493A** |
| **ΔΔG*_binding_*** | -0.0752 | 3.7284 | -2.9508 | -5.9192 | -0.0666 | 2.3026 | 0.1809 | -0.0510 | -0.3420 | -8.7765 |
| **ΔΔE*_vdW_*** | -0.015 | -0.319 | -6.047 | -1.600 | -0.060 | -5.929 | -0.134 | -0.035 | -0.038 | -2.340 |
| **ΔΔE*_EEL_*** | -0.009 | 210.404 | -1.481 | -4.977 | -0.537 | 1.559 | 0.322 | 0.435 | 0.410 | -30.003 |
| **ΔΔE*_EPS_*** | -0.051 | -206.311 | 5.027 | 0.695 | 0.530 | 6.981 | -0.002 | -0.451 | -0.714 | 23.844 |
| **ΔΔE*_ENPOLAR_*** | 0 | -0.0456 | -0.4498 | -0.0373 | 0 | -0.3087 | -0.0050 | 0 | 0 | -0.2782 |
| **Parameters** | **S494A** | **Y495A** | **F497A** | **Q498A** | **P499A** | **T500A** | **N501A** | **V503A** | **Y505A** |  |
| **ΔΔG*_binding_*** | -0.0975 | -0.5077 | -0.5164 | -15.6444 | -0.0582 | -2.5229 | 1.8483 | -0.2368 | -2.9706 |  |
| **ΔΔE*_vdW_*** | -0.045 | -0.207 | -0.157 | -1.774 | -0.213 | -1.122 | -2.382 | -0.381 | -6.011 |  |
| **ΔΔE*_EEL_*** | -3.413 | -2.213 | 0.157 | -24.871 | 0.165 | -7.842 | 3.928 | 0.295 | -11.258 |  |
| **ΔΔE*_EPS_*** | 3.361 | 1.912 | -0.516 | 11.156 | -0.008 | 6.585 | 0.279 | -0.056 | 14.914 |  |
| **ΔΔE*_ENPOLAR_*** | -0.0004 | 0 | 0 | -0.1568 | -0.0019 | -0.1436 | 0.0239 | -0.0944 | -0.6156 |  |

**Note:** The vdW and EEL represent van der Waals and the electrostatic contributions from MM, respectively. EPS stands for PB electrostatic contribution to the polar solvation free energy, while ENPOLAR is the nonpolar contribution to the solvation free energy. The binding energy changes (ΔΔG_binding_, kcal mol^-1^) are reported that demonstrates the relative affect the mutation in each the ΔG of binding complex, ΔΔG_binding_ = ΔG_wild-type_ – ΔG_alanine_.
